# The Roles of lncRNA in Cutaneous Squamous Cell Carcinoma

**DOI:** 10.3389/fonc.2020.00158

**Published:** 2020-02-28

**Authors:** Yujia Wang, Bensen Sun, Xiang Wen, Dan Hao, Dan Du, Gu He, Xian Jiang

**Affiliations:** ^1^Department of Dermatology, West China Hospital, Sichuan University, Chengdu, China; ^2^State Key Laboratory of Biotherapy, West China Hospital, Sichuan University, Chengdu, China

**Keywords:** cutaneous squamous cell carcinoma, lncRNA, ERK1/2, skin, cancer

## Abstract

Cutaneous squamous cell carcinoma derives from keratinocytes and is the second most common cause of non-melanoma skin cancer. Cutaneous squamous cell carcinoma (cSCC) develops rapidly and is also the leading cause of death in non-melanoma cancers. Lymph node metastasis occurs in 5% of cSCC patients, and some patients may even metastasize to the viscera. Patients with regional lymphatic metastasis or distant metastases have a <20% 10-year survival rate, indicating the substantial challenge in treating advanced and metastatic cSCC. Some lncRNAs have been found to be abnormally overexpressed in many tumor tissues, so that they can be considered as potential new biomarkers or targets that can be used in the diagnosis and treatment of cSCC in the future. In this review, we summarize the role of lncRNA in cutaneous squamous cell carcinoma to make a better understanding of mutations in cSCC and lay the foundation for effective target therapy of cSCC.

## Introduction

Skin is the largest organ of the human body composed of epidermis, dermis, and subcutaneous tissue. The epidermis belongs to the multilayer epidermal epithelium, which is mainly composed of keratinocytes. The outermost cuticle of the epidermis can protect against physical damage, chemical stimulation, and microbial invasion. However, a variety of risk factors such as chemicals, ultraviolet radiation, smoke, and pollutants, can cause skin damage and even skin cancer (1, 2). Cutaneous squamous cell carcinoma is derived from keratinocytes and is the second most common cause of non-melanoma skin cancer (3). The occurrence of cutaneous squamous cell carcinoma is an adverse outcome caused by the interaction of multiple factors, including environmental factors and self-factors. The most important risk factors for cSCC included skin color and age reversal of tumor suppressor genes such as *RAS, MYC, p53*, and *RUX3* (4). The adverse stimulation of the external environment is closely related to the occurrence of cSCC, such as sunlight exposure, chemical exposure, and viral infection. The occurrence of cSCC is generally believed that it is related to excessive ultraviolet irradiation (5). UV mainly causes DNA damage in cells, such as transformation of C-T and CC-TT pyrimidine dimer, activation of *p53*, and loss of function of regulating cell proliferation. In addition, mutations of *p53* and *RAS* have been found in patients with actinic keratosis induced by UV. It can be inferred that mutation of *p53* and *RAS* may be early changes of UV damage, which laid the foundation for the development of cSCC (6). Cutaneous squamous cell carcinoma develops rapidly, which is also the leading cause of death in non-melanoma cancers. Lymph node metastasis occurs in 5% of cSCC patients, and some patients may even metastasize to the viscera (7). Patients with regional lymphatic metastasis or distant metastases have a <20% 10-year survival rate, indicating the substantial challenge in treating advanced and metastatic cSCC (8). It has been reported that the down-regulation of *p21* mediated by melanoma-associated antigen A12 (*MAGEA12*) may be involved in the pathogenesis of cSCC, which indicates that *MAGEA12* might be a molecular biomarker of cSCC (9). And telomerase reverse transcriptase gene promoter (*TERTp*) mutation may be a molecular biomarker with prognostic significance for invasive cSCC, but further study is still needed (10). Meanwhile, Cortactin (*CTTN*) phosphorylation is closely related to the pathogenesis of cSCC and can be used as a molecular biomarker of cSCC (11). Positive pS6 seems to be the predictor of aggressiveness of cSCC combined with the operation history of cSCC, lesion-positive margin, degree of differentiation, and lesion size (12). The expression of cell division cycle 20 (*CDC20*) in cSCC tissues and cell lines increased significantly, which was related to the pathological differentiation of cSCC. It suggested that *CDC20* may be a new biomarker for the prevention diagnosis and treatment of cSCC (13). LncRNA is a transcript with a length of more than 200 nucleotides without open reading frame and does not encode proteins. It is capped at the 5′ end and polyadenylated at the 3′ end, and transcribed by RNA polymerase II. Compared to mRNA, the expression of lncRNA is not abundant, and it has poor conservativeness among species (14). Although most lncRNAs do not encode proteins, it has been reported that about 8% of lncRNAs in humans can encode short peptides; these results demonstrated that lncRNA regulates biological processes by encoding short peptides (15). Some lncRNAs have been found to have an abnormal overexpression in many tumor tissues, which can be considered as new potential biomarkers that can predict the canceration of tissues.

Generally, according to its relative position with coding genes, lncRNA can be basically divided into intergenic lncRNA, intronic lncRNA, sense lncRNA, antisense lncRNA, and bidirectional lncRNA. The location of lncRNA transcription in genomes often determines its related function (14, 16). In addition, it can be classified into four categories according to the function of lncRNA: signal, decoy, guide, and scaffold. LncRNA participated in the transmission of some signaling pathways when they acted as signals; some lncRNAs can regulate downstream gene transcription. As decoys, lncRNA can bind and remove some transcription factors or proteins to regulate gene expression. As guides, lncRNA can recruit cis- or trans-acting target genes of chromatin modifying enzymes. As scaffolds, lncRNA can bind a variety of proteins to form complex and modify histone in chromatin (17). As for the regulation mechanism of lncRNA, it can interact with DNA, RNA, or protein to regulate gene expression via various pathways. Firstly, lncRNA can be scaffolds or guides to regulate the related chromatin modifying enzymes in transcriptional processes (18). Secondly, lncRNA affected gene expression by regulating epichromatin modification (19). Thirdly, lncRNA regulated gene expression after transcription and regulated the level of mRNA and miRNA by different mechanisms such as competing endogenous RNA (20). Consequently, we reviewed the role of lncRNA in cutaneous squamous cell carcinoma to make a better understanding of mutations in cSCC and lay the foundation for effective target therapy of cSCC. It has been reported that the expression of lncRNA *MALAT1* was upregulated in tongue squamous cell carcinoma (TSCC) and was related to cervical lymph node metastasis. Further mechanism studies showed that *MALAT1* can inhibit tumor cell apoptosis and induce cell migration and invasion by regulating the Wnt/beta-catenin signaling pathway, and overexpression of *MALAT1* can induce epithelial mesenchymal transition (EMT) (21). Meanwhile, high *MALAT1* level was found in 54 cases of oral squamous cell carcinoma (OSCC) with poor prognosis (22). Compared with normal tissues, the expression of *HOTAIR* in OSCC was increased, which negatively correlated with E-cadherin level (23). And *HOTAIR* is highly expressed in TSCC, which was involved in the regulation of proliferation and apoptosis of TSCC (24). It was found that the expression of *GAS5* in OSCC was lower than that in normal tissues, suggesting that the overexpression of *GAS5* inhibited the proliferation, migration, and invasion of tumors (25).

## Role of lncRNA in Cutaneous Squamous Cell Carcinoma

### PICSAR

*PICSAR, LINC00162*, was a kind of p38 inhibited cutaneous squamous cell carcinoma associated with lincRNA, which was first reported in 2016. Piipponen et al. obtained cSCC cell lines from patients' skin tissue by surgical resection. By whole-transcriptome analyses, it has been found that several kinds of lncRNAs were differentially expressed in cSCC cell compared with primary NHEKs (normal human epidermal keratinocytes). Among them, long intergenic non-protein coding RNA 162 *(LINC00162)* was the most significantly up-regulated lncRNA. RNA *in situ* hybridization analysis showed that *PICSAR* was specifically expressed by tumor cells in cSCCs, but not by keratinocytes in normal skin *in vivo*. In addition, *PICSAR* played a carcinogenic role by regulating the mitogen-activated protein kinase/extracellular signal-regulated kinase (MAPK/ERK) signaling pathway, which is well-known to be dysregulated in cSCCs. It has been further determined that the carcinogenic mechanism of *PICSAR* was through inhibiting ERK2's negative regulator dual specificity phosphate 6 (DUSP6), while PICSAR can be inhibited by P38 MAPK. Besides, knockout of PICSAR suppressed the proliferation and migration of cSCC cells and inhibited the growth of human cSCC xenografts *in vivo* (26). Meanwhile, it has been mentioned that knockdown of *PICSAR* increased adhesion and decreased cell migration on collagen I and fibronectin by downregulating α2β1 and α5β1 integrin expression (27).

### TINCR

The gene of *TINCR* is located between *SAFB2* and *ZNRF4* genes on chromosome 19, which can promote epidermal differentiation through post-transcriptional mechanism. Studies show that the number of layers in human epidermal tissue layered granules decreased by 81.4%. Caspase decreased 83.7% in the absence of *TINCR*, which hydrolyzed protein and promoted apoptosis and is needed to maintain the function of the epidermal barrier (28, 29). Cutaneous squamous cell carcinoma derives from keratinocytes in the epidermis, which is closely related to epidermal differentiation. In human squamous cell carcinoma specimens, the expression of *TINCR* was down-regulated, which is consistent with the decrease in the differentiation of squamous cell carcinoma (28). Other research suggested that *TINCR* is involved in ALA-PDT-induced (5-aminolevulinic acid- photodynamic therapy-induced) apoptosis and autophagy in A431 cells. ALA-PDT promoted the expression of *TINCR* in A431 cells, and then TINCR promoted ALA-PDT-induced apoptosis and autophagy via the ERK1/2-SP3 (specificity protein 3) pathway (30).

### LINC00520

As a new type of lncRNA, *LINC00520* has been reported only in a few tumors. The expression of *LINC00520* increased in breast cancer. The oncogenes *SRC, PIK3CA*, and *STAT3* can regulate the expression of *LINC00520* and affect the progress of breast cancer (31). The expression of *LINC00520* was upregulated in laryngeal squamous cell carcinoma, and it was associated with lymph node metastasis (32). In cutaneous squamous cell carcinoma, *LINC00520* suppressed the invasion and metastasis of A431 cells via inhibiting EGFR and inactivating the PI3K-AKT signaling pathway (33).

### LINC00319

*LINC00319* was a newly discovered cancer-related lncRNA transcribed from the intergenic region of chromosome 21, which has been reported as a carcinogen in several human cancers, such as lung cancer (34), nasopharyngeal carcinoma (35), and ovarian cancer (36). It has been reported that *LINC00319* was significantly upregulated in cSCC tissues and cell lines. Functional studies showed that *LINC00319* promoted the proliferation of CSCC cells, accelerated the cell cycle process, promoted cell migration and invasion, and inhibited cell apoptosis. In mechanistic studies, *LINC00319* promoted cell proliferation, migration, and invasion through the regulation of CDK3 (cyclin-dependent kinase 3) in A431 cells mediated by miR-1207-5p (37).

### THOR

LncRNA *THOR* was a highly conserved long non-coding RNA mainly expressed in normal testis and tumors (38), which has also been shown to be closely related to the biological functions of tumors. For example, *THOR* can promote the proliferation of hepatocellular carcinoma cells and renal cancer cells, and even mediate cisplatin resistance in nasopharyngeal carcinoma (39–41). Knockdown of *THOR* in A431 cells downregulated *IGF2BP1*-dependent mRNAs, and then suppressed A431cell survival and proliferation. Targeting *IGF2BP1* by *Lnc-THOR* silencing might be a novel strategy to inhibit cSCCs (42).

### AK144841

*AK144841* is a new long noncoding found by Gilles et al., the expression of which in cSCCs was 40 times higher than that in healthy skin. *AK144841* was absent from normal keratinocytes, indicating that it may play a possible role in tumoral progression (43).

### MALAT1

*MALAT1*, a bona fide lncRNA, was highly transformed in mammals and widely expressed in human tissues (44) and played an important role in angiogenesis (45). *MALAT1* showed abnormally high expression in breast cancer (46), liver cancer (47), gastric cancer (48), and tongue squamous cell carcinoma (49), which promoted the migration and proliferation of cancer cells (50) and affected the drug resistance of cancer cells (51). In addition, it was related to the adverse prognosis of various solid tumors (52, 53). *MALAT1* was characterized to be highly expressed in cSCC tissues and cell lines. Zhang et al. established a novel c-MYC-assisted MALAT1-KTN1-EGFR axis. *MALAT1* regulated the protein expression of EGFR but did not affect the EGFR mRNA expression. Transcriptional sequencing identified KTN1 as the key mediator regulating EGFR. Mechanism studies have shown that *MALAT1* interacted with c-MYC to form a complex, which bound directly to the promoter region of KTN1 gene to enhance its activation, thereby actively regulating the protein expression of EGFR (54). Meanwhile, knocking down *MALAT1* significantly increased the protein expression of E-cadherin and β-catenin and decreased the protein expression of vimentin (55).

### LINC01048

According to data from the TCGA database, upregulation of intergenic length non-protein coding RNA 1048 (*LINC01048*) was associated with a low overall survival rate in cSCCs. Knockout of *LINC01048* inhibited cell proliferation and promoted cell apoptosis, suggesting the carcinogenic role of *LINC01048* in cSCCs. Mechanism studies showed that *LINC01048* increased the binding of TAF15 to the YAP1 promoter, thereby activating YAP1 in cSCC cells (56).

### GAS5

*GAS5*, a tumor suppressor (57), was usually induced by stress such as serum deficiency and cell-to-cell contact inhibition (58). The expression level of *GAS5* in renal, breast, lung, prostate, and bladder transitional cell carcinomas was significantly lower than that in normal tissues, which may lead to apoptosis avoidance and cell cycle disorder (57–62). Studies showed that *GAS5* in normal tissues was significantly higher than that in cSCC tissues. Overexpression of *GAS5* inhibited proliferation and promoted apoptosis in A431 cells (63).

### HOTAIR

**Figure 1:**
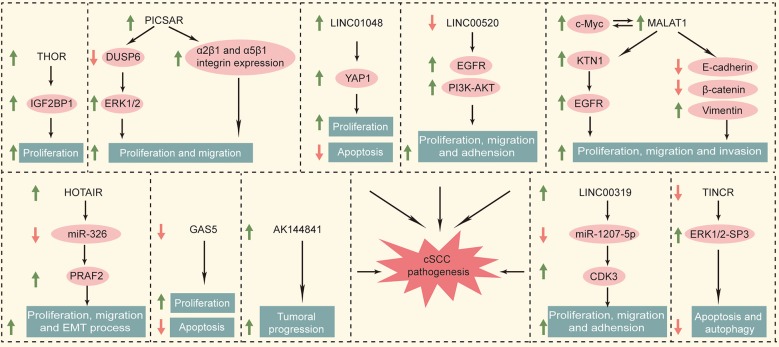
Functional roles of specific deregulated lncRNAs in cutaneous squamous cell carcinoma.

*HOTAIR* is the first lncRNA found to have a trans-acting effect (64). The *HOTAIR* gene sequence is less conserved except for specific regions, and evolved faster than the *HOXC* gene cluster located nearby (65). These two genes not only are closely related to the human embryonic development but also played a key regulatory role in the adult organ and tissue formation (66). *HOTAIR* is widely involved in the regulation of the malignant process such as proliferation, apoptosis, angiogenesis, invasion, and metastasis (67). *HOTAIR* monitored epigenetic modification by the histone H3K27me3 (68) and regulated WIF-1 and PTEN, thereby effecting the Wnt and Akt signaling pathways (69, 70). Another study showed that *HOTAIR* (HOX transcript antisense RNA) knockdown inhibited the motility and invasiveness of A375 cells and reduced the degradation of the extracellular matrix (71). Meanwhile, Liu et al. showed that overexpression of *HOTAIR* upregulated the PKR expression to activate PI3K/AKT and NF-κB pathways in keratinocytes (HACAT cells). This promoted the UVB-induced apoptosis and inflammatory injury (72). It has been shown that *HOTAIR* had higher expression levels in melanomas than in non-tumor tissues (73). It has been reported that the expression of *HOTAIR* in cSCC cells increased significantly. The overexpression of *HOTAIR* promoted the migration, proliferation, and epithelial–mesenchymal transitions by competitively combining with miR-326 to regulate the expression of PRAF2 (74). We summarized the functional roles of specific deregulated lncRNAs in cataneous squamous cell carcinoma in Figure 1.

## Conclusion

Cutaneous squamous cell carcinoma derives from keratinocytes and is the second most common cause of non-melanoma skin cancer. Lymph node metastasis occurs in 5% of cSCC patients, and patients with regional lymphatic metastasis or distant metastases have a <20% 10-year survival rate, suggesting that it is necessary to further understand the pathogenesis of cSCC. In this review, we summarized the role of lncRNA in cutaneous squamous cell carcinoma to make a better understanding of mutations in cSCC and lay the foundation for effective target therapy of cSCC. The expression of *PICSAR, LINC00319, THOR, AK144841, MALAT1, LINC10148*, and *HOTAIR* was upregulated in cSCCs. However, that of *TINCR, LINC00520*, and *GAS5* was downregulated in cSCCs. *MALAT1* and *HOTAIR* were upregulated in TSCC, OSCC, and cSCC, which may play a role in SCC diagnosis. Meanwhile, *GAS5* was downregulated in OSCC and cSCC. *PICSAR, LINC00319, THOR, AK144841, LINC10148, TINCR*, and *LINC00520* were only found in cSCC, which have a potential to be specific markers of cSCC. LncRNAs played different roles in cSCC, and we summarized them in Table 1 for a clearer understanding. Among them, *PICSAR* and *TINCR* regulated cSCC via the ERK1/2 pathway. We believed that there will be more lncRNA-related findings in future studies to improve cSCC therapy.

**Table 1:** List of lncRNAs currently implicated in cutaneous squamous cell carcinoma.

**LncRNA**	**C. location**	**Cells**	**Tissues**	**Expression in cSCCs**	**Confirmation *in vitro* and *in vivo***	**Related gene**	**Functional role**	**References**
PICSAR	Chr.21q22.3	Primary NHEKs	cSCC patients mice	Upregulated	qPCR and FISH verification in primary NHEKs and patients' tissues	p38/MAPK ERK1/2	Proliferation and migration	(26, 27)
TINCR	Chr.19p13.3	A431 cells		Downregulated	qPCR verification in A431 cells	ERK1/2-SP3	Apoptosis and autophagy	(28, 30)
LINC00520	Chr.14q22.3	A431 cells		Downregulated	qPCR verification in A431 cells	EGFR PI3K-AKT	Invasion and metastasis	(33)
LINC00319	Chr.21q22.3	SCL-1 cells A431 cells	cSCC patients	Upregulated	qPCR verification in A431 cells and SCL-1 cells qPCR and FISH verification in patients' tissues	CDK3	Proliferation, migration, and adhesion	(37)
THOR	Chr.2q14.2	A431 cells primary human skin SCC cells		Upregulated	qPCR verification in A431 cells and primary human skin SCC cells	IGF2BP1	Proliferation	(42)
AK144841	Chr.4qC.4	mSCC-20, mSCC-38-mSCC-20, and mSCC-38 cells A431 cells		Upregulated	qPCR verification in A431 cells			(43)
MALAT1	Chr.11q13.1	A431 cells, HSC-1 cells, HSC-5 cells		Upregulated	qPCR verification in A431 and HSC-1 cells. FISH verification in patients' tissues	c-MYC KTN1 EGFR	Proliferation, migration, and invasion	(54)
LINC01048	Chr.13q13.3	SCC13 cells, SCL-1 cells		Upregulated	qPCR verification in SCC13 and SCL-1 cells and patients' tissues	TAF15 YAP1	Proliferation and apoptosis	(56)
GAS5	Chr.1q25.1	A431 cells		Downregulated	qPCR verification in A431 cells		Proliferation and apoptosis	(63)
HOTAIR	Chr.12q13.13	A431cells, HSC-5 cells, SCC13 cells, and SCL-1 cells		Upregulated	qPCR verification in A431 cells, HSC-5 cells, SCC13 cells, and SCL-1 cells	miR-326 PRAF2	Migration, proliferation, and EMT process	(74)

## Author Contributions

YW, BS, GH, and XJ designed the study. YW, DH, DD, and XW performed the research. YW, BS, XW, and DH analyzed the data. YW, DD, GH, and XJ wrote the article.

### Conflict of Interest

The authors declare that the research was conducted in the absence of any commercial or financial relationships that could be construed as a potential conflict of interest.
